# Evaluation of hemodynamics in healthy term neonates using ultrasonic cardiac output monitor

**DOI:** 10.1186/s13052-020-00872-x

**Published:** 2020-08-05

**Authors:** Daniela Doni, Silvia Nucera, Camilla Rigotti, Elena Arosio, Valeria Cavalleri, Monica Ronconi, Maria Luisa Ventura, Tiziana Fedeli

**Affiliations:** 1 Neonatal Intensive Care Unit, FMBBM, San Gerardo Hospital, Monza, Italy; 2 Università Milano Bicocca, Milan, Italy

**Keywords:** Neonatal hemodynamics, USCOM, Transition phase, Cardiovascular adaptation

## Abstract

**Background:**

Transition from intrauterine to extrauterine life is a critical phase during which several changes occur in cardiovascular system. In clinical practice, it is important to have a method that allows an easy, rapid and precise evaluation of hemodynamic status of a newborn for clinical management. We here propose a rapid, broadly applicable method to monitor cardiovascular function using ultrasonic cardiac output monitoring (USCOM).

**Methods:**

We here present data obtained from a cohort of healthy term newborns (*n* = 43) born by programmed cesarean section at Fondazione MBBM, Ospedale San Gerardo. Measurements were performed during the first hour of life, then at 6 + 2, at 12–24, and 48 h of life. We performed a screening echocardiography to identify a patent duct at 24 h and, if patent, it was repeated at 48 h of life.

**Results:**

We show that physiologically, during the first 48 h of life, blood pressure and systemic vascular resistance gradually increase, while there is a concomitant reduction in stroke volume, cardiac output, and cardiac index. The presence of patent ductus arteriosus significantly reduces cardiac output (*p* = 0.006) and stroke volume (*p* = 0.023). Furthermore, newborns born at 37 weeks of gestational age display significantly lower cardiac output (*p* < 0.001), cardiac index (*p* = 0.045) and stroke volume (*p* < 0.001) compared to newborns born at 38 and ≥ 39 weeks. Finally, birth-weight (whether adequate, small or large for gestational age) significantly affects blood pressure (*p* = 0.0349), stroke volume (*p* < 0.0001), cardiac output (*p* < 0.0001) and cardiac index (*p* = 0.0004). In particular, LGA infants display a transient increase in cardiac index, cardiac output and stroke volume up to 24 h of life; showing a different behavior from AGA and SGA infants.

**Conclusions:**

Compared to previous studies, we expanded measurements to longer time-points and we analyzed the impact of commonly used clinical variables on hemodynamics during transition phase thus making our data clinically applicable in daily routine. We calculate reference values for each population, which can be of clinical relevance for quick bedside evaluation in neonatal intensive care unit.

## Background

Physiologically, the transition from intrauterine to extrauterine life is a critical phase, in particular for the cardiovascular system. The transition from fetal to neonatal circulation starts at delivery with umbilical cord clamping, and it is characterized by major cardiovascular changes, occurring within seconds to hours. However, it takes longer to complete the transition, as definitive anatomical closure of ductus arteriosus can take several weeks or even months. Fetal circulation allows the left ventricle to work with low resistance. At delivery, there is a rapid and remarkable increase in pressure in left ventricle and a concomitant decrease of pressure in the right ventricle [1].

It appears clear that cardiovascular adaptation to extrauterine life is a complex process, with important consequences on newborn health. It is important for physicians to have a rapid and direct measure of the ongoing process in order to early diagnose life-threatening pathologies. Several methods have been proposed to monitor cardiac output in term and preterm newborns [2, 3]. Invasive methods are considered gold standard to measure cardiac output (e.g. Swan-Ganz catheter) as for accuracy but are of limited applicability in newborns. Among non-invasive techniques, echocardiography is the most widely used. However, it requires well-trained personnel and the use of an expensive instrument, which limits its widespread applicability.

The ultrasonic cardiac output monitoring (USCOM) was introduced in 2001 as a method to measure hemodynamic parameters. In particular, it allows to measure trans-thoracically Doppler flow within large vessel. USCOM is a non-invasive and easy-to-learn technique. By measuring Doppler flow in big vessels (e.g. aorta) the algorithm calculates cross-sectional area of the aortic tract based on validated normograms derived from height and weight. The flow in ascending aorta, related to blood pressure and aortic valve area, provides information about cardiovascular function (such as cardiac output, cardiac index and systemic vascular resistance). USCOM is compact and cheaper than conventional ultrasound machine and it is designed for users with no prior ultrasound experience. USCOM has been widely used on adult patient; there are several studies on pediatric population, but data on newborns are still few. In order to be clinically applicable, normal reference values must be established for newborns as was done for other populations (adults, children) [4]. A Chinese group published some years ago a study in which they monitored cardiac output in healthy term infants during transitional phase, starting from 4 h after birth up to 8 h of life [5]. In a recent work USCOM was used on newborn in comparison to echocardiography and it was shown to over-estimate CO [6]. In this study we aimed to confirm previously published data on healthy newborn population but extending the analysis to longer time-points, and we aimed to assess the impact of several commonly found conditions on the transition phase.

## Materials and methods

We performed a perspective single center study with the aim of establishing reference values of the Italian newborn’s hemodynamic parameter during the transition from fetal to post-natal circulation using ultrasonic cardiac output monitoring system (USCOM®Pty Ltd., Sydney, Australia).

We enrolled healthy term newborn born at San Gerardo Hospital- “Fondazione Monza e Brianza per il Bambino e la sua Mamma” after having acquired written parents’ informed consent, in accordance with ethical institutional rules. All newborns were > 36 + 6 gestational week and were born by planned caesarian section from January 2017 to May 2019.

Exclusion criteria for enrollment were: congenital heart disease, need of resuscitation at birth, respiratory distress (any grade), denial of consent.

We assessed blood flow in ascending aorta by measuring flow at suprasternal notch through a compact ultrasound probe (diameter of 12 mm). USCOM records blood flow through aortic valve by mean of continuous doppler wave (2,2 MHz) and obtain the velocity time integral (VTI). An anthropometric algorithm, based on length and weight, calculates aortic cross-sectional area (CSA). The product of CSA times VTI is stroke volume (SV). Using measurement of heart rate (from the distance between systolic peaks of the flow), USCOM software obtains cardiac output (CO) as follows: SV x heart rate = CO. Cardiac index (CI), was determined as cardiac output (SV x HR) with respect to body surface.

To complete cardiovascular assessment, we measured systolic, diastolic and mean blood pressure at each time-point using a DINAMAP Procare 100 (GE Medical Systems) with neonatal arm cuff. Information about afterload was recorded as systemic vascular resistance (SVR) and systemic vascular resistance index (SVRI): systemic vascular resistance related to cardiac output and blood pressure (BP/CO) with respect to body surface.

USCOM was performed at four time-points: during the first hour of life (time-point 1), then at 6 + 2 (time-point 2), 12–24 (time-point 3), and 48 h of life (time-point 4). We performed an echocardiography to identify a patent duct at 24 h and, if patent, it was repeated at 48 h of life.

While performing USCOM examination, babies were supine, preferably quiet, and the time of evaluation was quick (less than 5 min).

Statistical analysis was performed using Graphpad Prism (version 5.0). Normal range was calculated as 95% Confidence interval.

## Results

**Fig. 1:**
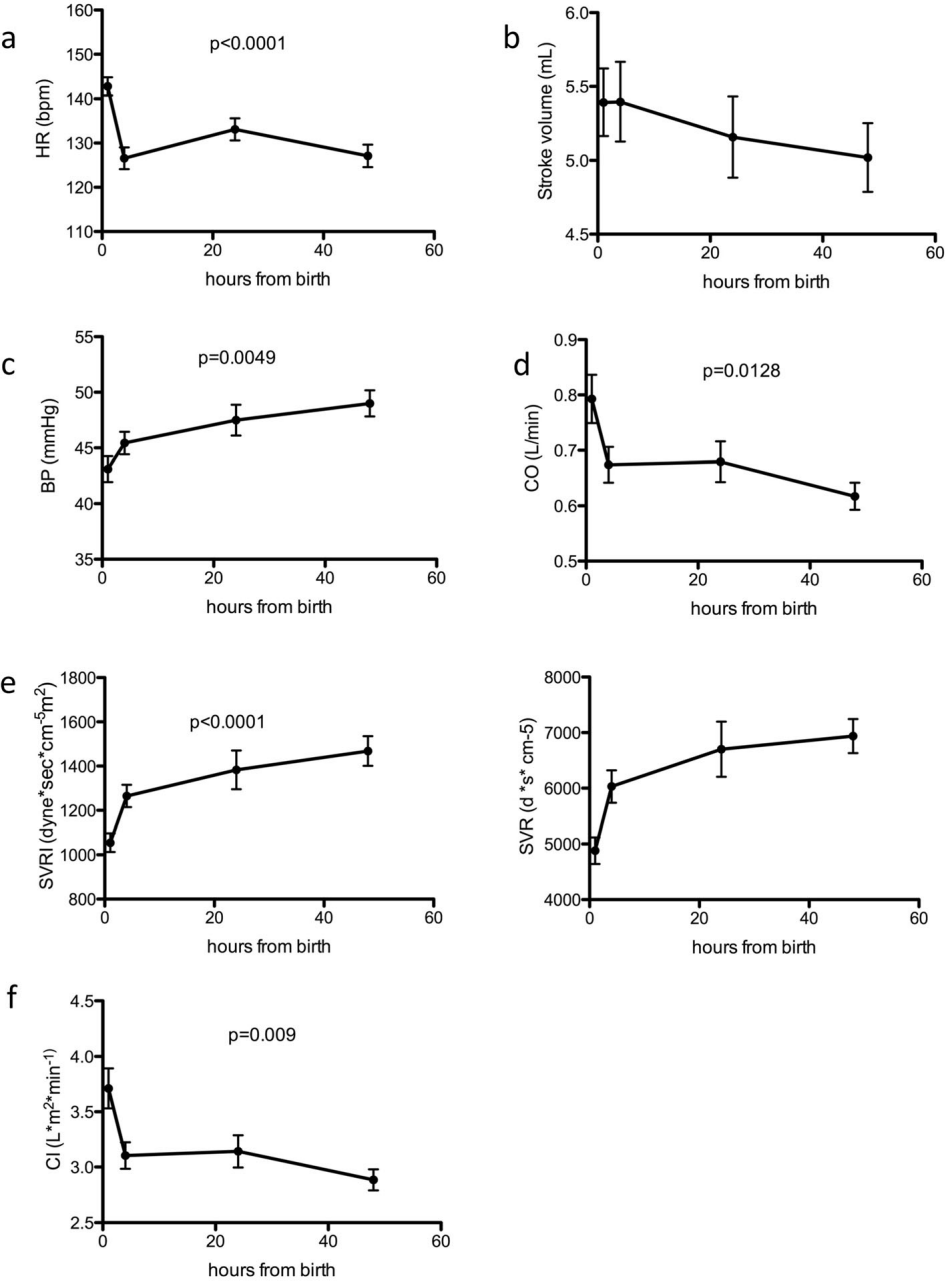
(**a**) Heart rate distribution at different time points (**b**) Stroke volume at different time points. (**c**) Mean blood pressure at different timepoints. (**d**) Cardiac output at different time points. (**e**) SVRI at different timepoints. (**f**) Cardiac index at different timepoints. Analysis was performed using Kruskal-Wallis test with Dunn’s multiple comparison. 95% CI

Our sample population was composed by 43 healthy term newborns born by programmed cesarean section (see Table 1 for population characteristics). The population was characterized by mean gestational age of 38 + 3 weeks (range 37–41) and the mean birth weight was 3.15 ± 0.58 kg (range 1.92–4.27 Kg). The majority of the sample is appropriate for gestational age (72% AGA, *n* = 31), 14% SGA (*n* = 6), 14% LGA (*n* = 6). Nineteen out 43 were females (44%) and 24 out 43 were males (56%). We compared heart rate (HR), stroke volume (SV), mean arterial blood pressure (BP), cardiac output (CO), systemic vascular resistance (SVR), indexed SVR (SVRI) and cardiac index (CI) at each time point (Fig. 1a-f and Table 2). We found statistically significant modifications of HR, BP, CO, SVRI and CI over time. In particular, SVRI increased over time, while CI, stroke volume and CO decreased after the first 4–6 h of life (Fig. 1a-f and Table 2). Stroke volume was the only index that did not show statistically significant modifications.

**Table 1:** Table depicts characteristic of our sample population

Population characteristics	
***Sex***	
**Male**	*n* = 19 (44%)
**Female**	*n* = 24 (56%)
***Origin***	
**Italian**	*n* = 32 (74%)
**Other European**	*n* = 4 (9%)
**Non European**	*n* = 7 (16%)
***Weight at birth***	
**AGA**	*n* = 31 (72%)
**LGA**	*n* = 6 (14%)
**SGA**	*n* = 6 (14%)
***Gestational age***	
**37 weeks** (37 + 0–37 + 6)	*n* = 12 (28%)
**38 weeks** (38 + 0–38 + 6)	*n* = 16 (37%)
**≥ 39 weeks**	*n* = 15 (35%)
***Arterious duct***	
**Patent**	*n* = 14 (33%)
**PDA > 48 h**	*n* = 6 (14%)
**Not patent**	*n* = 29 (67%)

**Table 2:** The table depicts the value of RSVI, CI, HR, SV and CO in our sample population. Mean, maximum, minimum and normal range as 95% CI are reported

	Mean	Max	Min	Normal range	
RSVI (d*s*cm^−5^/m^2^)					
1 h	1042,1	1974,8	515,7	935	1103
4–6 h	1264,8	2062,9	638,8	1166	1363
24 h	1382,8	3218,5	453,4	1211	1555
48 h	1468,3	3121,2	827,5	1417	1680
CI (l/min/m^2^)					
1 h	3,7	8,6	2,3	3,33	4,06
4–6 h	3,1	5,3	1,7	2,87	3,33
24 h	3,1	6,2	1,2	2,81	3,39
48 h	2,8	4,7	1,6	2,61	2,99
HR (bpm)					
1 h	143	174	106	138	147
4–6 h	127	162	97	122	132
24 h	133	182	95	128	138
48 h	127	152	77	116	126
SV (cm^3^)					
1 h	5,6	12,6	3,0	5,04	6,16
4–6 h	5,4	9,2	2,8	4,9	5,9
24 h	5,2	8,3	1,8	4,68	5,72
48 h	5,0	8,7	2,4	4,56	5,45
CO (L/min)					
1 h	0,8	1,8	0,4	0,71	0,89
4–6 h	0,7	1,0	0,3	0,64	0,76
24 h	0,7	1,2	0,2	0,63	0,77
48 h	0,6	0,9	0,3	0,55	0,65

After having assessed normal values of cardiovascular parameters and their modifications over time, we decided to analyze the impact of other clinical factors. In particular, we decided to analyze the influence of PDA, birth-weight (whether appropriate, small or large for gestational age) and gestational age (GA).

**Fig. 2:**
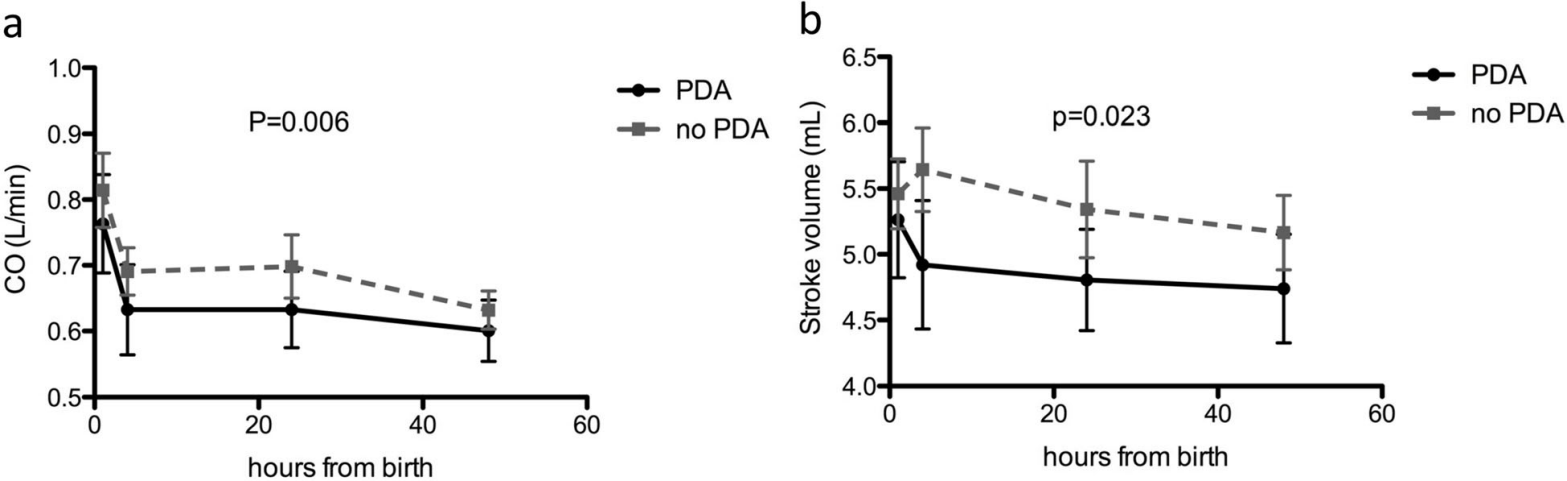
(**a**) Cardiac output significantly differs according to the presence of PDA. Paired t test. (**b**) Stroke volume is also affected by the presence of PDA. Paired t test

As aforementioned, we performed a screening cardiac ultrasound to assess presence of patent ductus arteriosus. PDA was present at 24 h of life in 14 out of 43 analyzed patients, corresponding to 33%. According to gestational age 41% of newborns at 37 weeks of gestation have PDA; 44% at 38 weeks of gestation and 30% after 39 weeks of gestation have PDA respectively. There is no significant difference in the distribution of PDA according to gestational age (1 way ANOVA, Kruskal-Wallis, Figure S1a). We therefore analyzed the impact of PDA on cardiovascular transition phase. We found that the presence of PDA significantly affects CO and Stroke volume (Fig. 2a and b), with no effect on other parameters (Figure S1b-e). We calculated normal values for our population of Stroke volume and CO according to the presence of PDA (Table 3).

**Table 3:** The table reports SV and CO for newborns with PDA and without PDA. Mean, maximum, minimum and normal range as 95% CI are reported

	PDA normal range		Non PDA normal range	
SV (cm^3^)				
1 h	4,38	6,22	4,97	6,03
4–6 h	3,9	5,9	4,96	6,24
24 h	4,04	5,6	4,58	6,02
48 h	3,89	5,51	4,63	5,77
CO (L/min)				
1 h	0,64	0,96	0,69	0,91
4–6 h	0,44	0,76	0,63	0,78
24 h	0,49	0,71	0,59	0,81
48 h	0,49	0,71	0,53	0,68

**Fig. 3:**
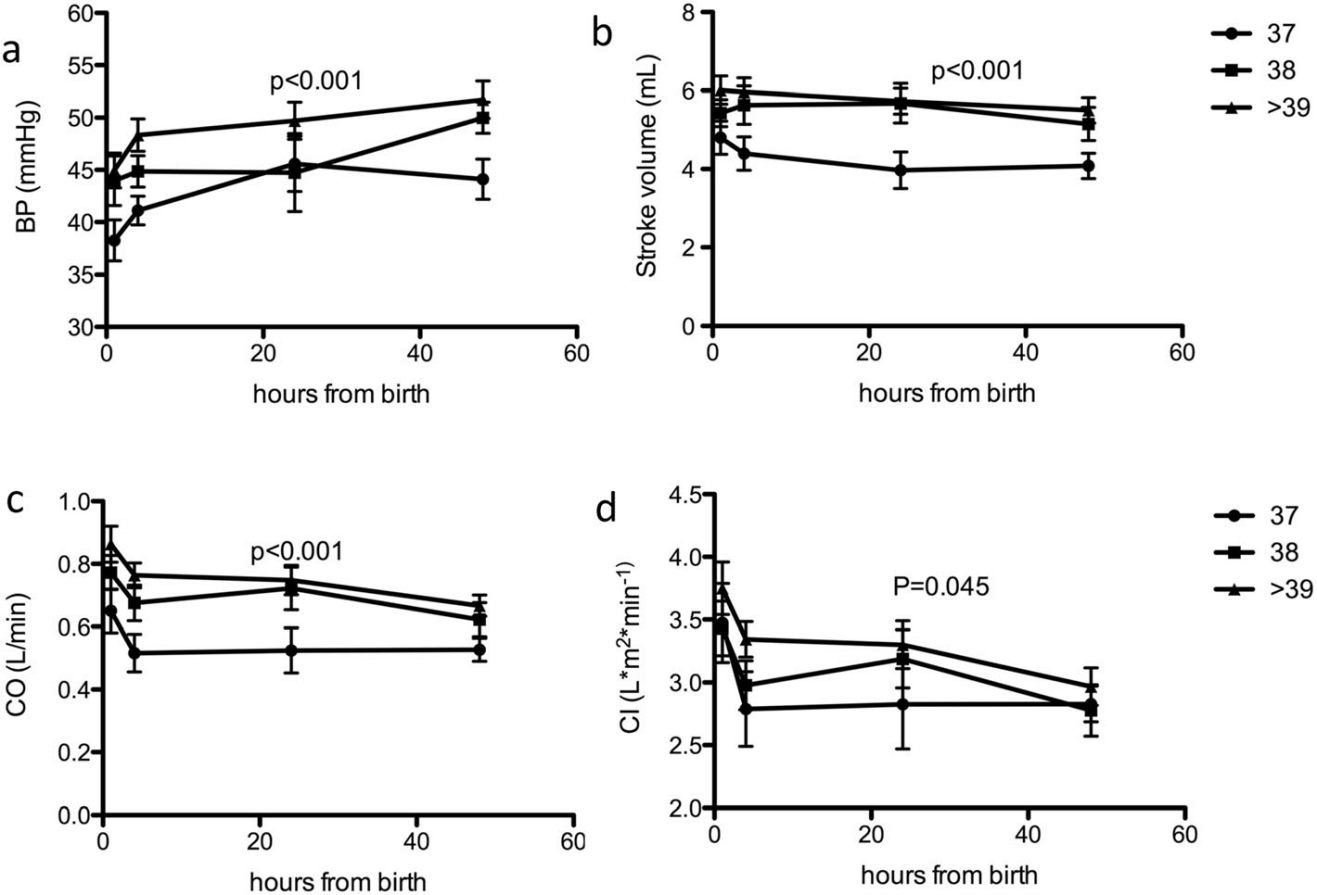
(**a**) Effect of gestational age on Mean Arterial Blood Pressure, in particular of gestational age of 37 weeks. 2 Way ANOVA with Bonferroni correction. (**b**) Effect of gestational age on Stroke volume, in particular of gestational age of 37 weeks. Newborns at 37 EG display significantly lower stroke volume compared to later term newborns. 2 Way ANOVA with Bonferroni correction. CI 95% (**c**) Effect of gestational age on Cardiac Output, in particular of gestational age of 37 weeks. Newborns at 37 EG display significantly lower cardiac output compared to later term newborns 2 Way ANOVA with Bonferroni correction. CI 95% (**d**) Effect of gestational age on Cardiac Index, in particular of gestational age of 37 weeks. 2 Way ANOVA with Bonferroni correction. CI 95%

Subsequently, we decided to analyze whether the cardiovascular parameters changed according to gestational age. Interestingly, we found that BP, CO, SV and CI were significantly affected by gestational age. Newborns at 38 and ≥ 39 weeks of gestation (range 39 + 0–41 + 5) behaved the same, while those born at 37 weeks behave differently (Fig. 3a-d and Table 4).

**Table 4:** The table reports BP, SV, CO and CI according to gestational age. Mean, maximum, minimum and normal range as 95% CI are reported

	37 EG normal range		38 EG normal range		≥ 39 EG normal range	
BP (mmHg)						
1 h	34,35	42,05	38,63	47,78	41,7	48,29
4–6 h	38,39	43,8	41,96	47,84	45,2	51,4
24 h	40,4	50,8	37,8	52,02	46,15	53,25
48 h	40,3	47,9	47	52,94	48,15	55,25
SV (cm^3^)						
1 h	3,98	5,6	4,72	6,08	5,3	6,7
4–6 h	3,6	5,2	4,67	5,3	5,3	6,7
24 h	3,08	4,92	4,71	6,7	5,04	6,36
48 h	3,45	4,75	4,39	6	4,84	6,16
CO (L/min)						
1 h	0,59	0,81	0,68	0,92	0,77	1,03
4–6 h	0,39	0,61	0,58	0,82	0,71	0,89
24 h	0,39	0,61	0,58	0,82	0,61	0,79
48 h	0,44	0,56	0,48	0,72	0,61	0,79
CI (l/min/m^2^)						
1 h	2,88	4,12	2,97	3,83	3,31	4,09
4–6 h	2,23	3,37	2,63	3,38	3,04	3,56
24 h	2,12	3,48	2,77	3,63	2,91	3,7
48 h	2,52	3,08	2,43	3,17	2,69	3,31

**Fig. 4:**
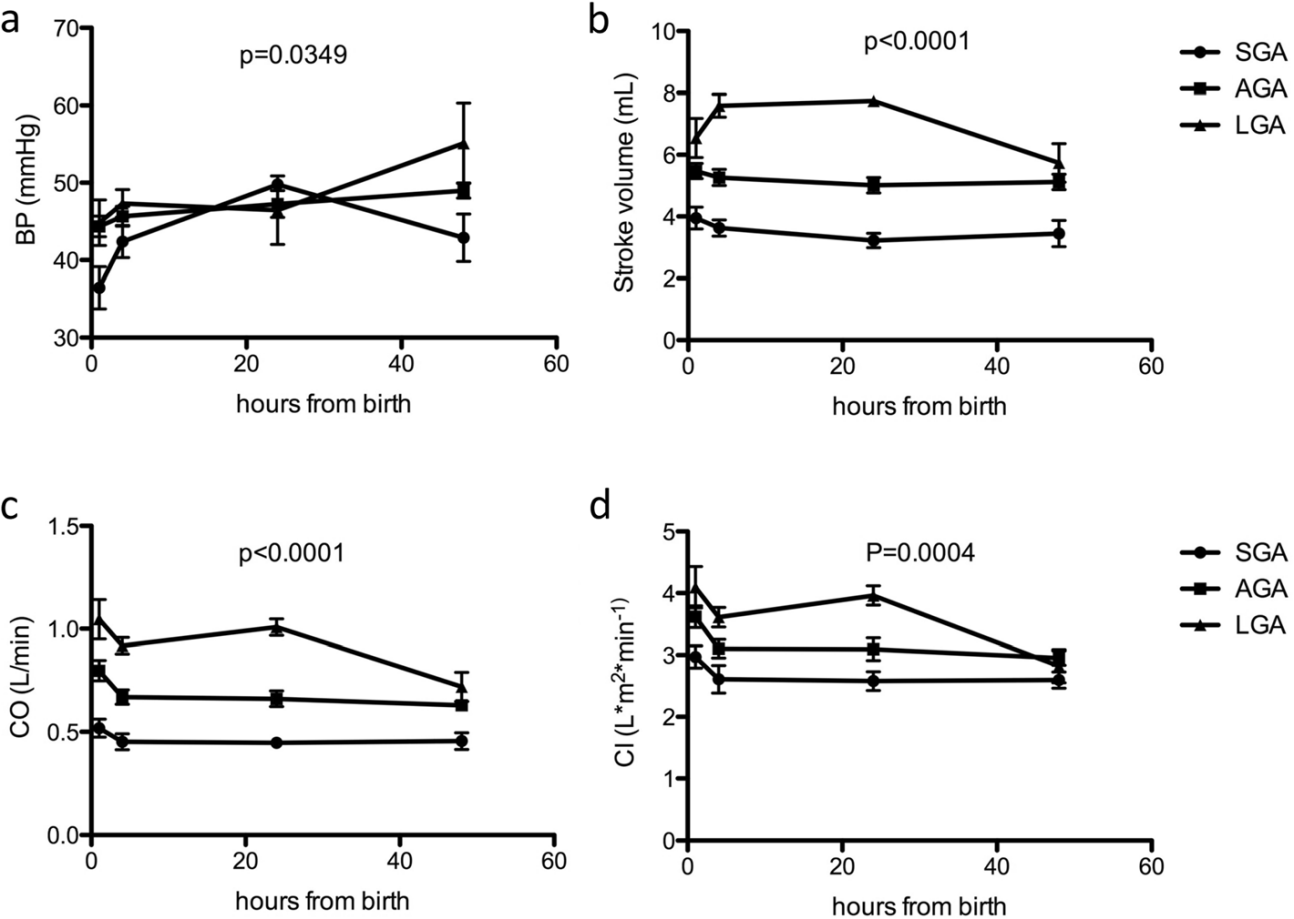
(**a**) Effect of birth weight on BP. 2 Way ANOVA with Bonferroni correction. CI 95% (**b**) Effect of birth weight on Stroke volume. 2 Way ANOVA with Bonferroni correction. (**c**) effect of birth weight on cardiac output. 2 Way ANOVA with Bonferroni correction. 95% CI (**d**) effect of birth weight on cardiac index. 2 Way ANOVA with Bonferroni correction. 95% CI

Finally, we analyzed the impact of birth-weight by distinguishing AGA, SGA and LGA. In our dataset 6 out of 43 newborns were LGA (14%), which corresponds to percentages reported in literature. On the other hand, SGA newborns may have had intra-uterine growth restriction (IUGR), which also affects developing cardiovascular system. Alternatively, SGA newborns (defined as birth weight < 10° percentile according to the week of gestation) may have underlying genetic conditions which will be diagnosed later in life and therefore constitute a separate group from AGA newborns. In our dataset we had 6 out of 43 infants who were defined as SGA, corresponding to 14%. We found significant differences in BP, SV, CO and CI (Fig. 4a-d and Table 5). In particular, concerning SV, CO and CI, LGA newborns displayed a different behavior from AGA and SGA. For SV, after an initial increase in LGA infants, it decreased at 48 h, while AGA and SGA remained stable after the first 4–6 h of life. As for CO the initial behavior was the same but it markedly decreased at 48 h in LGA infants. CI slightly increased in LGA infants at 24 h then decreased. In summary, we show that SV, CO and CI of LGA newborns, while initially higher than AGA and SGA newborns, tend to normalize at 48 h of life while AGA and SGA newborn reach a stable value already at 4–6 h of life.

**Table 5:** The table reports BP, SV, CO and CI according to weight at birth. Mean, maximum, minimum and normal range as 95% CI are reported

	SGA normal range		AGA normal range		LGA normal range	
BP (mmHg)						
1 h	31,04	41,76	41,79	47,01	39,04	50,56
4–6 h	38,32	46,48	43,12	48,28	44,32	51,68
24 h	48,36	51,24	43,73	50,67	36,08	55,12
48 h	36,9	48,9	47	51	50,2	67
SV (cm^3^)						
1 h	3,18	4,62	5,04	5,97	5,7	8,1
4–6 h	2,88	4,32	4,76	5,84	7,06	8,34
24 h	2,72	3,68	4,5	5,5	7,56	8,04
48 h	2,6	4,2	4,6	5,6	4,66	6,74
CO (L/min)						
1 h	0,42	0,58	0,69	0,91	0,84	1,16
4–6 h	0,42	0,58	0,63	0,77	0,82	0,98
24 h	0,32	0,48	0,63	0,77	0,92	1,08
48 h	0,42	0,58	0,56	0,37	0,54	0,86
CI (l/min/m^2^)						
1 h	2,68	3,32	3,24	3,96	3,46	4,74
4–6 h	2,2	3	2,81	3,39	3,28	3,92
24 h	2,28	2,92	2,74	3,5	3,68	4,32
48 h	2,36	2,84	2,75	3,25	2,24	3,36

## Discussion

In our study, we present data from a population of healthy newborns obtained by using USCOM during transition phase. The aim of the study was to assess the changes of cardiovascular parameters in the first 48 h of life. The relevance of this study is to create reference values that could be used in clinical setting to evaluate acutely ill newborns. These data are in line with previously published data [5] although in previously published data there were no time-points after 8 h of life. In our study, we expanded the analysis to longer time-points (up to 48 h after birth) in order to cover the whole transition phase. Moreover, we also extended the analysis taking into account several clinically relevant parameters such as weeks of gestation, presence of PDA or appropriateness of birth-weight. Our aim is to underline that these conditions, in particular the appropriateness of birth-weight, besides being used to classify newborns, really reflect a different biologic/physiologic background.

As shown in Fig. 2 and Figure S1, PDA reduces CO and stroke volume without affecting the other parameters, this is probably due to a compensatory mechanism of increasing of SVR (Figure S1e) although in our dataset the effect is not statistically significant, maybe due to the small size of the sample. These data are apparently in contrast with previously published data [7]; however, it is also important to underline that in our dataset, PDA was detected via screening ultrasound performed on all newborns, and it is not hemodynamically significant.

Another interesting finding concerns gestational age. In our dataset, newborns born at 37 weeks of gestation behave differently from newborns from 38 on weeks of gestation. This is in line with findings on neonatal acute respiratory distress syndrome, which show that newborns at 37 weeks of gestation behave more like late-preterm infants rather than full term. Altogether, these finding suggest that from a biological standpoint, it should be appropriate to change the definition of full term infants to > 38 weeks of gestation [8].

LGA newborns (defined as birth weight > 90° percentile according to the week of gestation) are well known to have an increased number of neonatal complications including hypoglycemia and respiratory distress [9, 10]. Furthermore, LGA infants are often born from diabetic mothers and the impact of maternal diabetes on fetal heart is well known as it is known the higher incidence of cardiac malformations in this cohort [11]. In our dataset only 1 out of 6 LGA was born from diabetic mother (gestational diabetes). However, it is important to underline that during postnatal adaptation phase, there are major changes in cardiomyocyte metabolism. Some reports in literature show alterations of placental vascular tone in LGA newborns born from obese mothers [12]. In particular, it is reported that Nitric Oxide (NO) production is reduced in chorionic vessels from LGA newborns compared to AGA counterpart. This is the consequence of reduced adiponectin levels on obese mothers compared to non-obese mothers and an impaired response to adiponectin. It has also been reported that maternal obesity is associated with increased vascular resistance in human umbilical arteries [13] and with reduced chorionic vessel relaxation in response to oxidative stress [14], suggesting that maternal obesity severely affects NO-dependent vascular function in feto-placental unit. This may impact on cardiovascular function of newborn and may thus explain the different behavior in our dataset of LGA newborns compared to AGA and SGA counterparts. However, in our dataset, we found no significant differences in body mass index (BMI) of the mothers before pregnancy. There is however a trend towards a higher BMI of mothers of LGA infants (data not shown). Whether this may explain the differences in cardiovascular parameters is still to be clarified. Our data are in line with a recently published paper which uses USCOM to compare a cohort of term newborns with low-birth-weight newborn and very low birth weight newborns [15]. In this work, the authors show that parameters such as CO, SV and CI proportionally increase with birth weight, compatible with what we observed in our dataset for SGA and LGA (Fig. 4b-d). In this work, however, there are no data on LGA infants so it is not possible to directly compare these data with our results.

Despite being widely used on adult patients, USCOM applicability to newborn is still debated. In particular it is debated how much USCOM measures agree with traditional echocardiography measure in determining cardiac output with contrasting data in literature [5, 6, 16, 17]. Recently published data on newborns show that USCOM may over-estimate CO compared to echocardiograph. However, the authors confirm that USCOM may be reliable if used for repetitive measures or if appropriate corrections are made [6]. In our study, we show that USCOM may be considered reliable since we find concordance between measures by USCOM and echocardiography for what concerns PDA patients. As aforementioned, USCOM is noninvasive, easily applicable, and requires short users training time. These characteristics make USCOM a good candidate to be readily available in delivery room and in nurseries to quickly monitor at risk newborns. The presence of reference values, even if not derived from large population studies, may help in clinical setting. Our study is a preliminary study performed on a small, which has to be confirmed by subsequent studies with an increased number of newborns. As a preliminary study, we chose to analyze only newborns from elective cesarean section in order to obtain data from a possibly homogeneous population. It will be important to include newborns born from vaginal delivery in future studies. However, our study paves the way for larger population studies that can establish more robust reference values, in particular for relatively rare conditions such as PDA or LGA/SGA newborns.

In conclusion, we assessed using USCOM how cardiac function changes during transition phase and how different conditions can affect it. We need to increase our sample size and we will in future concentrate on specific patient populations. However, we here propose a pilot study that may be valuable for many neonatal care units.

## Supplementary information

**Additional file 1: Figure S1.** (a) Distribution of PDA (1 = PDA, 0 = no PDA) in newborns according to gestational age. (b) Apart from CO and SV, the other parameters are not significantly different in newborns with PDA. The figure shows how SVRI is not affected by the presence of PDA. (c) CI is not affected by PDA. (d) BP is not affected by PDA. (e) SVR is not affected by PDA.

## Data Availability

The datasets used and/or analysed during the current study are available from the corresponding author on reasonable request.

## Abbreviations

HR: Heart rate
SV: Stroke volume
BP: Mean arterial blood pressure
CO: Cardiac output
SVR: Systemic vascular resistance
SVRI: Indexed SVR
CI: Cardiac index
PDA: Patent ductus arteriosus
AGA: Adequate for gestational age
SGA: Small for gestational age
LGA: Large for gestational age
