# Supplementary figures and images for: Evaluation of hemodynamics in healthy term neonates using ultrasonic cardiac output monitor
Source: Ital J Pediatr. 2020 Aug 5;46:112. doi: 10.1186/s13052-020-00872-x (PMC7405450; doi:10.1186/s13052-020-00872-x)

# Supplementary Figure 1

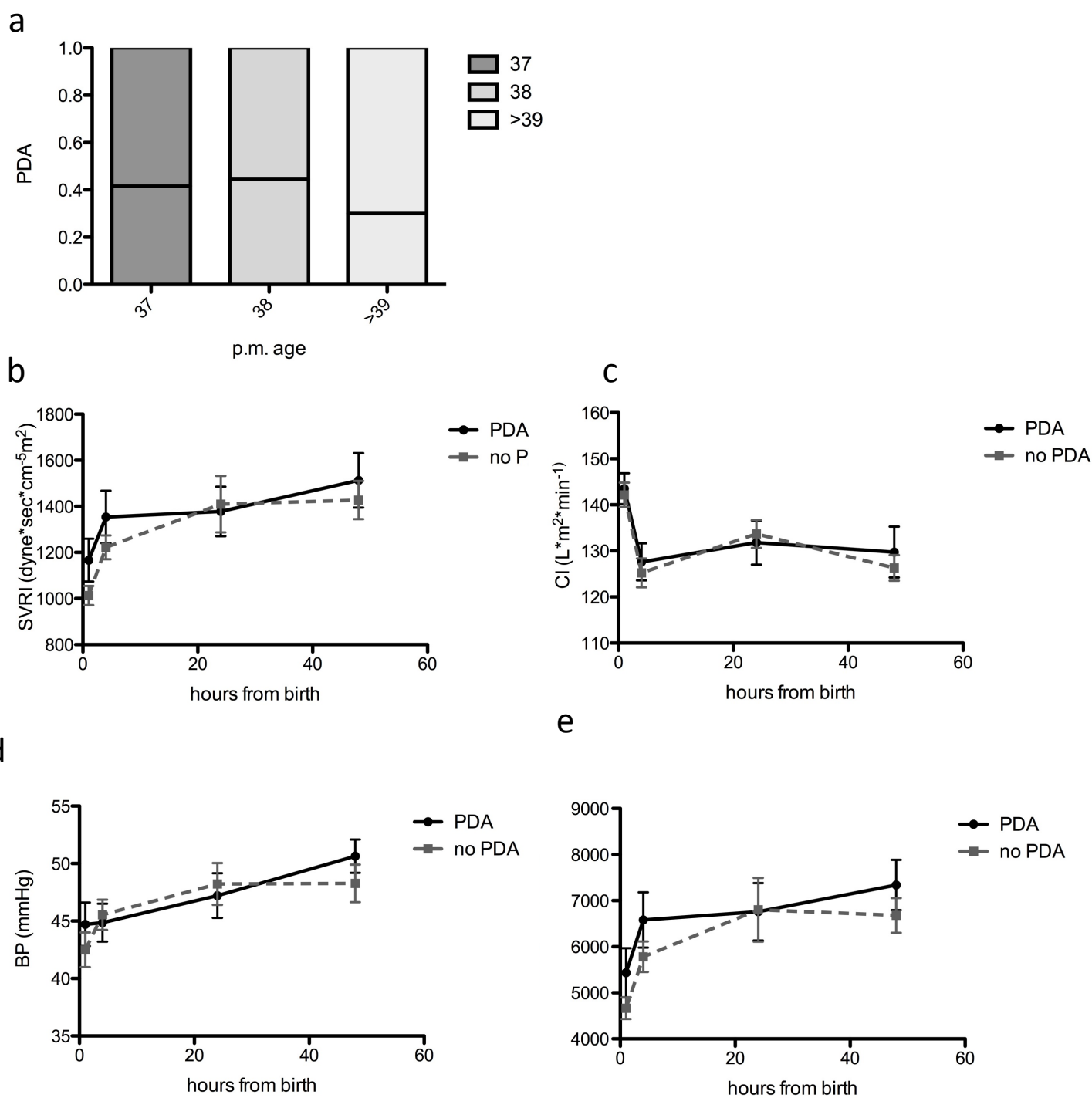

Supplement: Supplementary file 1 — Additional file 1: Figure S1. (a) Distribution of PDA (1 = PDA, 0 = no PDA) in newborns according to gestational age. (b) Apart from CO and SV, the other parameters are not significantly different in newborns with PDA. The figure shows how SVRI is not affected by the presence of PDA. (c) CI is not affected by PDA. (d) BP is not affected by PDA. (e) SVR is not affected by PDA. [file 13052_2020_872_MOESM1_ESM.pdf]
